# With great power comes great responsibility – deductions of using ChatGPT for medical research

**DOI:** 10.17179/excli2023-6163

**Published:** 2023-06-12

**Authors:** Samar Mahmood, Mohammad Omer Khan, Ishaque Hameed

**Affiliations:** 1 Department of Internal Medicine, Dow University of Health Sciences, Baba-e-Urdu Road, Karachi, Pakistan

## ⁯⁯

This letter explores the risks of using ChatGPT for medical research, while duly acknowledging its many benefits. On one hand, ChatGPT can provide quick and accurate analysis of large amounts of medical data, making it a valuable tool for medical researchers. However, there are various ethical concerns surrounding the use of AI in medical research, including issues related to data privacy, biases, authenticity of generated content and accountability. This letter also discusses the need for transparency and accountability in AI-based medical research to ensure that the results are reliable and trustworthy. In conclusion, the use of ChatGPT for medical research offers great potential for advancing medical knowledge. However, it is important to approach this technology with caution and to constantly evaluate its merits, demerits and limitations. This 'great power' comes accompanied by a responsibility to lay some strict ground rules. By doing so, we will be able to ensure that AI is used in a responsible, regulated and ethical manner in the field of medical research and in all others. 

The introduction of Open AI's free tool, ChatGPT, came as a revolutionary advancement in the realm of artificial intelligence in November 2022 (Open AI, 2022[9]). As a development that seemed to shake the internet's tectonic plates, it caused a sense of instant polarization amongst early adopters, some with largely positive feedback and others raising concerns about its possible misuses and controversies (Gao et al., 2023[5]). As a large language model (LLM) with a complex neural network base that has the ability to generate tone- and content-defined, coherent output, ChatGPT is built on Generative Pre-trained Transformer-3 (GPT-3)-trained with 175 billion parameters (Gao et al., 2023[5]; Baruffati, 2023[2]). Attracting 57 million users in its first month post-launch and building to an estimated average of 96 million visitors per month as of March 2023, ChatGPT has certainly made its concrete place in the online world (Baruffati, 2023[2]). Like in all other disciplines, the influence of ChatGPT in medicine is limitless as well, with currently discovered areas including clinical decision support, medical record keeping and translation, interpretation of imaging, medication management, disease surveillance, telemedicine, clinical trial recruitment, patient triage, drug information, mental health support, remote patient monitoring, medical education, and research (Marr, 2023[8]).

Similar to writers in all other fields, ChatGPT came as a promising superpower for medical researchers too. It was described as an efficient tool that catalyzed productivity while reducing the required human effort (Sallam, 2023[10]). The language and expressivity-enhancing capability of the AI tool was welcomed as a benefit, specifically for non-native English speakers, and extrapolated to potentially promote equity and diversity in medical research (Sallam, 2023[10]). However, as its use spread among researchers and the initial elation of its convenience settled in, the spotlight turned towards its limitations. An extensive systematic review of 60 records that examined ChatGPT in the context of health care education, research, or practice reported the model's generation of superficial, incorrect, or inaccurate content as well as its extraction of non-existent, 'made up' references as two of the most glaring problems that threatened research quality (Sallam, 2023[10]). An analysis of some of these fake references showed that they were made up of 'plausible components' - including real author names strung together as co-authors, authentic journal names with mismatched year ranges, and occasionally matching volume and issue numbers with mostly inaccurate page numbers (Hillier, 2023[6]). The provided PubMed IDs and digital object identifiers were also nonexistent. These 'literature salads' have been described as having such a high degree of plausibility that they would be 'a trap for unwary users' and require utmost vigilance in reviewing (Hillier, 2023[6]).

'Artificial hallucinations,' as the above errors are labeled, are claimed by ChatGPT itself as "not common in chatbots, as they are typically designed to respond based on pre-programmed rules and data sets rather than generating new information. However, there have been instances where advanced AI systems, such as generative models, have been found to produce hallucinations, particularly when trained on large amounts of unsupervised data" (Alkaissi and McFarlene, 2023[1]). These confident, blatant blunders are just the first of the problems with the use of ChatGPT in medical research. As an answer to a question posed to the AI model itself, it enlisted six of its prominent limitations in medical research, namely: lack of domain-specific knowledge, limited ability to interpret visual data, difficulty in managing complex data sets, restricted capacity to handle causation vs. correlation, dependence on data quality, and need for significant computing power (Answer retrieved on ChatGPT, May 08 2023). Moreover, as crucially highlighted by Dahmen et al., further issues include those of plagiarism and a lack of contextual data that paves the way for inaccuracies and racial as well as sexist biases (Dahmen et al., 2023[3]; Homolak, 2023[7]). The inability of the AI model to understand the nuances related to medical science and language can lead to serious underestimation and overlooking of articles on important issues that are fewer in number (Dahmen et al., 2023[3]). To add to this, when given a mixture of original and AI-generated abstracts, blinded human reviewers incorrectly identified 14 % of original abstracts as being generated (Gao et al., 2023[5]). Generated abstracts seemed to bypass regular plagiarism detectors too, showing 100 % originality. Even the specific AI detector scored 34 % of generated abstracts in a way to imply a lesser probability of being unoriginal (Gao et al., 2023[5]).

What, then, should be done? To expect that the use of ChatGPT and other AI models will remain minimal after reading articles like this one would be ignorant. To minimize the above-mentioned perils, it is important to first be aware of them while using the tool and to advise responsible, clear disclosure when a manuscript is written with assistance from ChatGPT (Gao et al., 2023[5]). As patterns of AI output detection are being enhanced and work is ongoing to embed watermarks in outputs, running submissions through AI output detectors as part of the research editorial process could be an instrumental step taken by journal editorial boards (Gao et al., 2023[5]). Strict guidelines should be implemented by the journals regarding AI use in scholarly papers to restrict its misuse (Dave et al., 2023[4]). On the part of the researchers, using ChatGPT within the bounds of brainstorming, proofreading, and editing and as an aid for all other tasks strictly as an adjunct under their own supervision would be a safe step forward, so as to hold accountability in the face of any ethical dilemmas (Homolak, 2023[7]). Furthermore, for the developers of this powerful, state-of-the-art technology, honing its ability to either accurately conduct a literature search and withdraw authentic articles and citations, or otherwise deny requests that are not within its current capacity, would increase transparency and build user trust.

In conclusion, in order to harness the complete potential of ChatGPT in the medical and scientific fields, cautious implementation with controlled usage is imperative, along with the promotion of widespread 'AI literacy' (Hillier, 2023[6]). A persistent and transparent discourse about its advantages and drawbacks would be more beneficial than impulsive endorsement or excessive reliance.

## Declaration

### Acknowledgments

None.

### Conflict of interest

None.
